# The Biology of the Future

**Published:** 1888-01-14

**Authors:** W. E. Fothergill


### The Biology of the Future.

By W. E. Fothergill, M.A.

The most frequent topic of conversation for professional people and laymen alike has been for some weeks the new Life of Charles Darwin. Accordingly the present moment is a suitable one for a glance at the subject to which the "greatest man of the century" devoted his life. Such a glance will show his striking superiority over any of his followers, and will teach that the present duty of biologists is to ignore most of the work of these, and, retracing their mental footsteps to the point where Darwin left them without a leader, to begin seeking thence a new path for science. We have been led off on the wrong scent since Darwin left the pack, and we must hark back in due humility, taking care, however, not to go further than is necessary, for people who are so very eager to get to the beginnings of things often never get there at all, though they move backwards all their lives. In tracing back the history of biology, any violent efforts would be quite unrewarded. The dawn of the science, according to the custom of dawns, is darkly shrouded. I once began trying to unveil it, and referred to a celebrated author who shall be nameless, out of consideration for such of my readers as shall fail to recognise him. The first description I found was that of a great and strange beast, who, having no joints, was unable to lie down, and, having fallen, could not rise again. When exhausted nature claimed her due, he was constrained to prop himself up against some tree of the forest, and sleep all night thus leaning. The natives of that country, with the usual sapiency of the savage mind, were accustomed to discover the tree whereon the beast was fain to lean, and to cut it half through during the day. The tree thus weakened was wont to yield in the night under the weight of the beast, so that the natives found him lying helpless beside its fallen trunk in the morning, and having slain him, they fed on his meat many days.

Lest the charge of amphibiology be put upon me instead of on my author, I will refrain from further citations, and without describing more particularly the biology that considered every animal a sort of god, pass to the much more recent view that God created each species quite apart from every other one. The "special creation" hypothesis, this belief in a separate act of creation for every plant and every animal, still lingers in some lay minds; but it calls for no attention in considering the opinions of recent biologists. It slowly became clear to scientific men that our universe of suns and stars, our own earth with its vegetal and animal life, arrived at its present state through an infinite number of infinitely small changes, and not through sudden revolutionary ones. The geologists showed how natural forces like wind and moving water have gradually determined the various sorts of rocks, and how the characters of these have given its form to the surface of our earth. Their discoveries of fossil remains of plants and animals confirmed suspicions, already formed, that our living species are the results of small changes always going on, and caused by the same physical forces as those which act in the non-living world, but working in a more complete manner.

It is popularly supposed that Mr. Darwin discovered "evolution," and that evolution means that man descended from ape-like progenitors by natural selection of the biggest and strongest monkeys, who always beat their weaker brethren in the battle of life, and survived to perpetuate the race.

Let us look at the facts of the case. Aristotle was the first to conceive the possibility of the slow modification of plants and animals from smaller numbers and simpler conditions into their present complexity. Darwin was the thirty-seventh recognisable evolutionist, and since the publication of his book on the Origin of Species we have lost count. The notion of the production of one race from another by a series of small changes has always been repulsive to theologists and others, and they have held various more or less remarkable beliefs directly opposed to, or in some degree compromising with, those of the scientists of their times; but these, again, have no interest for us, as we trace through a few generations of thinkers their successive scientific positions.

When it was recognised that changes are always going on in plants and animals by which the individuals of a race vary amongst themselves, and by whose summation in the past the differences between races have arisen, some explanation of the cause of these changes was naturally demanded. The first explanation offered was that of Lamarck, who called attention to the fact that when an organ is used it grows, and when allowed to fall into disuse it dwindles away, and finally vanishes altogether. The circumstances in which living beings are placed are, of course, always varying, and the changing conditions demanding corresponding changes in the organism, these were supposed to arise, and to be increased or lost by use and disuse. Mr. Darwin mentions, as an illustration of this, the fact that the leg bones are larger in domestic ducks than in wild ducks, and the bones of the wing are smaller in proportion. This, of course, is because the ducks that became tame have used their legs more and their wings less than their wild ancestors, and the wild children of these. Everyone knows that a man can increase the size and power of particular muscles by exercising them, and that children resemble their parents in peculiarities acquired by the excessive use of certain parts. It was thought again that giraffes had gained long necks by stretching them continually, generation after generation, to crop the higher leaves of trees. Indeed all the forms of plants and animals were explained as the results of use and disuse.

But only a few of these explanations were satisfactory, and the principle failed altogether to account for many structures. Thorns, for example, are very useful to plants in protecting them from animals which eat the softer ones and leave the prickly to grow. But thorns get no exercise in not being eaten, and if they do happen to be devoured that cannot possibly increase their growth; so use and disuse can have nothing to do with the development of thorns. Anyone can think of equally striking examples—such as the colours of flowers and animals; and, indeed, it is difficult to find a structure of which the principle affords a complete explanation.

Naturally, then, it was completely superseded when Mr. Darwin and Russell Wallace called attention to the principle called by them "natural selection."

Giraffes all have long necks, they said, because in the struggle for food between their ancestors giraffes with necks longer than the others could reach more leaves, and so lived where others died. Their young had long necks like their parents, and of these the tallest were again naturally selected, and so on. With regard to the thorns on certain plants, it is easy to see how the softest members of a slightly thorny species would be eaten, and the thorniest left alone to produce thorniest seedlings, of which again the thorniest survived, till all the living members of the species became thorny.

Natural selection, however, is the selection of what? Of variations in individuals advantageous to them in some way or other, and enabling them to outdo their neighbours in the struggle for existence. How, then, do such variations arise? Why had some plants the beginnings of thorns, and how came it about that some giraffes had longer necks than others? This part of the subject Mr. Darwin leaves in mystery. He gives a force which can take advantage of favourable variations, but about the origin of these and the laws of variation he has very little to say. Some changes in the organism are clearly produced directly by the action of food and surroundings. But most of them Mr. Darwin leaves unexplained, saying that at present means are not available for the discovery of their laws and causes.

Now, it is clear that to know about these variations of individuals is as important as to understand the way in which they are exaggerated into the differences between species and genera. But though Mr. Darwin himself called attention to this, and made many investigations in this direction, most of his followers have concentrated their whole attention on the other, the easier side of the subject, and assumed that we can never know anything about variations and their causes, but that they must be considered spontaneous or fortuitous, and indefinite. More than this, they have raised natural selection from the position that Mr. Darwin expressly gave it as an important factor, but not the sole one, in organic evolution. They have considered it to the almost entire exclusion of the other forces acting. For the last twenty years "Natural Selection," "The Survival of the Fittest" (Mr. Herbert Spencer's equivalent), and "The Struggle for Existence," have been on all tongues. To every question the answer has been the same, whether the facts of the case were explicable by reference to the great principle or not. Natural selection has been deified, and is continually referred to by biologists, just as creation used to be invoked by theologians. The biology of the present is, putting it briefly, the application of the principle of natural selection to the solution of all questions, whether it bear upon the facts of the case or not.

But natural selection is felt to be insufficient. Public opinion is being prepared to receive further developments of the science, and the more philosophic of scientific men are silently ignoring the tirades of their contemporaries, and are retracing their steps to the point whence Darwin started. They are studying the laws of variation as well as the forces that take advantage of it. Definite causes are being assigned to changes, and definite rules are being found that guide their progress. Thus it appears, as Herbert Spencer showed several months ago, that the direct influence of its surroundings on the organism accounts for far more than has hitherto been attributed to it. Lamarck's principle has been dug up and revivified, and "use and disuse" promises to relieve natural selection of many weights it has hardly supported alone. Indeed, knowledge of the laws of variation shows that many facts are more clearly explained without reference to natural selection than they can be by its aid, so that the theory of evolution would be largely strengthened through the removal of severe strains from its as yet solitary stay.

The principle of survival of the fittest being brought down from its undue elevation and ranged where Mr. Darwin puts it as one important factor amongst others in organic evolution, we see that struggle for individual advantage is not the only raising force, but that sacrifice of the individual for the race is an even stronger one. This reconciles our higher feelings and our knowledge of mankind with our knowledge of the lower animals, and removes that formidable objection to the evolution theory sometimes known as the sentimental one.

To enter into the technical establishment of the argument would demand a distinct paper, and those interested will not be satisfied with anything but the full treatment of the case, and this they will find elsewhere.* We see, however, that biological science cannot progress till its forces bear their true relations, and even form a solid front to advance to the conquest of the unknown. To arrive at this state of things, natural selection, and the notion of struggle which has so long filled the minds of all, must give way till the subject has been viewed equally in all lights. The biologist must no longer look at the creature dead on the dissecting-table, but borrow for a time the eyes of the physician, and explain physiological changes as outcomes of temperament and constitution. He must see that the forms of living beings are due to tendencies inherent in their nature. How they have acquired these is a still deeper question, and demands a still further investigation of the action of its surroundings on the organism. Meanwhile, we may safely predict that the biology of the immediate future will be the science of variation.

* See Herbert Spencer: "Factors of Organic Evolution" (reprint from *Nineteenth Century*, May, 1886); and Proceedings of Linnean Society, papers by Patrick Geddes on "Variation," 1886, 1887; also papers to the British Association, Section Biology, on the same subject.

